# Influenza A virus infection and cigarette smoke impair bronchodilator responsiveness to β-adrenoceptor agonists in mouse lung

**DOI:** 10.1042/CS20160093

**Published:** 2016-04-10

**Authors:** Chantal Donovan, Huei Jiunn Seow, Jane E. Bourke, Ross Vlahos

**Affiliations:** * Biomedicine Discovery Institute, Department of Pharmacology, Monash University, Clayton, Victoria 3800, Australia; † Lung Health Research Centre, Department of Pharmacology and Therapeutics, University of Melbourne, Parkville, Victoria 3010, Australia; ‡ School of Health and Biomedical Sciences, RMIT University, Bundoora, Victoria 3083, Australia

**Keywords:** acute exacerbations of COPD (AECOPD), β-adrenoceptor, chronic obstructive pulmonary disease (COPD), influenza, respiratory virus, small airways

## Abstract

β_2_-adrenoceptor agonists are the mainstay therapy for patients with asthma but their effectiveness in cigarette smoke (CS)-induced lung disease such as chronic obstructive pulmonary disease (COPD) is limited. In addition, bronchodilator efficacy of β_2_-adrenoceptor agonists is decreased during acute exacerbations of COPD (AECOPD), caused by respiratory viruses including influenza A. Therefore, the aim of the present study was to assess the effects of the β_2_-adrenoceptor agonist salbutamol (SALB) on small airway reactivity using mouse precision cut lung slices (PCLS) prepared from CS-exposed mice and from CS-exposed mice treated with influenza A virus (Mem71, H3N1). CS exposure alone reduced SALB potency and efficacy associated with decreased β_2_-adrenoceptor mRNA expression, and increased tumour necrosis factor α (TNFα) and interleukin-1β (IL-1β) expression. This impaired relaxation was restored by day 12 in the absence of further CS exposure. In PCLS prepared after Mem71 infection alone, responses to SALB were transient and were not well maintained. CS exposure prior to Mem71 infection almost completely abolished relaxation, although β_2_-adrenoceptor and TNFα and IL-1β expression were unaltered. The present study has shown decreased sensitivity to SALB after CS or a combination of CS and Mem71 occurs by different mechanisms. In addition, the PCLS technique and our models of CS and influenza infection provide a novel setting for assessment of alternative bronchodilators.

## Abstract (precis)

The present study has shown that the bronchodilator effectiveness of β-adrenoceptor agonists is diminished in CS and influenza A virus-induced lung disease and has identified the need for the development of novel bronchodilators for these diseases.

## CLINICAL PERSPECTIVES

• CS and respiratory virus infections are a major cause of COPD and exacerbations of COPD, respectively. The aim of the present study was to assess the effects of β-adrenoceptor agonists on changes in small airway reactivity using mouse PCLS prepared from CS-exposed mice and from CS-exposed mice infected with influenza A virus.
• This is the first study to demonstrate impaired β-adrenoceptor sensitivity of small airways in PCLS from mice exposed to CS and mice exposed to CS infected with influenza A virus.
• We provide a validated model to assess novel bronchodilator agonists that may be of use in CS-induced lung disease such as COPD and influenza A virus-induced exacerbations of COPD.

## INTRODUCTION

Small airway dysfunction plays a crucial role in many obstructive lung diseases including chronic obstructive pulmonary disease (COPD) [1,2]. Cigarette smoking is a major contributor to COPD and also alters immune function to increase susceptibility to subsequent influenza A virus infection [3]. These infections are a major cause of acute exacerbations of COPD (termed AECOPD) [4].

AECOPD are defined by a sudden worsening of symptoms such as dyspnoea, wheezing, sputum production and cough initiated by bacterial, viral or environmental agents, usually leading to hospitalization [5,6]. Of note, infection with influenza virus is associated with greater inflammatory responses [7] and loss of lung function compared with exacerbations due to other causes [8]. The greater severity of disease following infection in smokers compared with non-smokers [9,10] is associated with inflammation and injury extending to the smaller airways in the distal lung [11] and increased influenza-related mortality [12,13].

A further contributor to disease severity is the marked decrease in bronchodilator efficacy of β-adrenoceptor agonists in AECOPD [14]. The mechanisms and pathways involved in this loss of responsiveness to therapy following cigarette smoke (CS) exposure and viral-induced exacerbations remain poorly understood [15]. Therefore, preclinical models of COPD have been developed to explore the mechanisms underlying CS-induced lung inflammation and viral-induced exacerbations (as reviewed in [16,17]).

To date, the use of precision cut lung slices (PCLS) to characterize *in vitro* changes in small airway reactivity in these contexts has been limited. We have recently shown that *in vivo* exposure to CS for 4 days altered contractile responses of airways in PCLS to serotonin (5HT), but not methacholine (MCh), but dilator responses were not assessed [18]. Previous studies using PCLS to assess the effects of viral infection alone have been limited to assessment of inflammation or infectivity after *in vitro* exposure [19,20]. In one study, PCLS from C57BL/6 mice were treated with poly I:C, a toll-like receptor 3 (TLR3) agonist structurally similar to viral dsRNA [19]. Increased release and tissue levels of tumour necrosis factor α (TNFα), interleukin-6 (IL-6) and monocyte chemoattractant protein 1 (MCP-1) confirmed a marked inflammatory response of structural cells and/or resident immunocytes. In another study, PCLS from pigs were exposed *in vitro* to five different types of swine influenza virus [20]. The swine influenza viruses infected both the ciliary cells and the mucous producing cells in the epithelium, with the highest titration of virus at 24 h post-infection. However, these studies of viral exposure *in vitro* did not assess airway contraction or relaxation. Therefore, it is clear from the literature that the effects of *in vivo* viral infection in any animal species with or without exposure to CS, on *in vitro* airway reactivity have not been explored.

Mem71 is a reassortment of influenza virus strain carrying haemagglutinin of strain A/Memphis/1/71 (H3) and neuraminidase of strain A/Bellamy/42 (N1). We have recently shown that mice exposed to CS for 4 days and then infected with Mem71 on day 5, had an exacerbated airway inflammatory response compared with either stimulus alone [21]. Moreover, prior CS exposure disrupts the resolution of influenza infection in mice. However, the influence of CS alone or the combination of CS and Mem71 exposure *in vivo* on bronchodilator responses remains to be addressed.

Given this background, the aim of the present study was to assess the effects of the β_2_-adrenoceptor agonist salbutamol (SALB) on changes in small airway reactivity using mouse PCLS prepared from CS-exposed mice and from CS-exposed mice treated with influenza A virus (Mem71, H3N1).

## MATERIALS AND METHODS

### Materials

All materials are from Sigma–Aldrich unless otherwise stated.

### Animals and ethics

All experimental procedures were approved by the Animal Ethics Committee at the University of Melbourne (approval number 1212485). Male mice were obtained from the Animal Resources Centre, Western Australia aged 6–12 weeks. All mice were acclimatized for a minimum of 3 days prior to beginning experimental protocols and randomly assigned treatment groups. Each group of mice (sham alone, CS alone for 4 days or sham PBS, sham Mem71, CS PBS, CS Mem71 over 12 days) were housed in separate cages with a maximum of ten mice per cage, and allowed standard animal chow and water *ad libitum*.

### CS exposure

Mice were placed in an 18 L Perspex chamber and exposed to CS generated from nine Winfield Red cigarettes (comprises <16 mg tar, <1.2 mg nicotine and <15 mg of carbon monoxide) per day for 4 days as described above [18,22]. Sham mice were exposed to room air for the same time periods. Mice were culled on day 5, and lungs were collected and used for either preparation of PCLS or for quantitative real-time PCR after freezing in liquid nitrogen.

### CS exposure followed by Mem71 infection

Mice were exposed to CS or room air (for sham) for 4 days (as described above). On day 5, mice were anaesthetized by inhalation of methoxyflurane (Medical Developments International) and infected intranasally with 10^4.5^ plaque forming units (PFU) of the mildly virulent influenza A virus Mem71 (H3N1) in a 30 μl volume, diluted in PBS as described [21]. Control mice were anaesthetized as described above and given 30 μl of PBS. All mice (sham PBS, sham Mem71, CS PBS, CS Mem71) were weighed daily throughout the protocol between 8:30 and 8:45 hours, and culled on day 12 (7 days post-infection or PBS). Lungs were collected and used for either PCLS or quantitative real-time PCR.

### Precision cut lung slices preparation and protocols

PCLS were prepared as recently described [18,23]. Briefly, mice were killed by overdose of 0.4 mg/ml of 60 mg/ml sodium pentobarbitone, injected i.p. Lungs were inflated with ∼1.4 ml of 2% agarose gel dissolved in 1X HBSS (hanks balanced salt solution) containing 20 mM Hepes (Gibco/Invitrogen) followed by a bolus of air through the trachea via a two port 20G cannula (Intima). Lungs were solidified in ice-cold 1X HBSS/Hepes before slices (150 μm) were serially sectioned from the left lobe using a vibratome (VT 1000S, Leicamicrosystems) and transferred into a 24-well plate containing Dulbecco's Modified Eagle's Medium (DMEM) supplemented with 1% penicillin–streptomycin solution (Gibco/Invitrogen). Slices were incubated at 37°C and 5% CO_2_ for subsequent experimental use.

Experiments were performed within 48 h of PCLS preparation. For contractile agonists MCh, 5HT, each concentration of drug was perfused for 5 min. For bronchodilator experiments, airways were submaximally pre-contracted with 5HT prior to cumulative additions of SALB or isoprenaline (ISO) at 5 min intervals.

### Quantitative real-time PCR

Lungs were prepared as described above [18]. Gene expression was quantitatively analysed using an ABI Prism 7900HT sequence detection system (Life Technologies) using a pre-validated TaqMan® Gene Expression Assays. The primer ID number for the β-adrenoceptor (gene: *adrb2*): Mm02524224_s1, 18S rRNA: Mm03928990_g1, gene: *IL-1β*: Mm00434228_m1, muscarinic M_2_ receptor (gene: *chrm2*): Mm01701855_s1, muscarinic M_3_ receptor (gene: *chrm3*): Mm00446300_s1, gene: *TNF*: Mm00443260_g1.

### Statistical analysis

All PCLS data are expressed as mean ± S.E.M. For contractile agonists, results are expressed as % initial airway lumen area, and for β-adrenoceptor agonists as % relaxation, with each *n* representing one slice per mouse. Paired *t* tests, unpaired *t* tests, one-way ANOVA with Dunnett's or Bonferroni's *post-hoc* tests were performed on potency and maxima obtained from fitted individual curves as outlined in the figure legends. Body weight data were expressed as change in grams from the starting weight, with statistical analysis by one-way ANOVA with *post-hoc* tests on raw weight values over time within group and at day 12 between groups. For quantitative real-time PCR analysis, threshold cycle (*C*_T_) values were generated for each sample and analysed using the ΔΔ*C*_T_ method relative to the internal control gene (*18S rRNA*). All data are expressed as median plus interquartile range and compared using Mann–Whitney non-parametric test compared with PBS or one-way ANOVA compared with sham PBS where appropriate. *P*<0.05 was used to indicate statistical significance. Statistical analysis was carried out on Graph Pad Prism™ (version 5.0b).

## RESULTS

### CS exposure impairs bronchodilator responses to salbutamol

**Figure 1:**
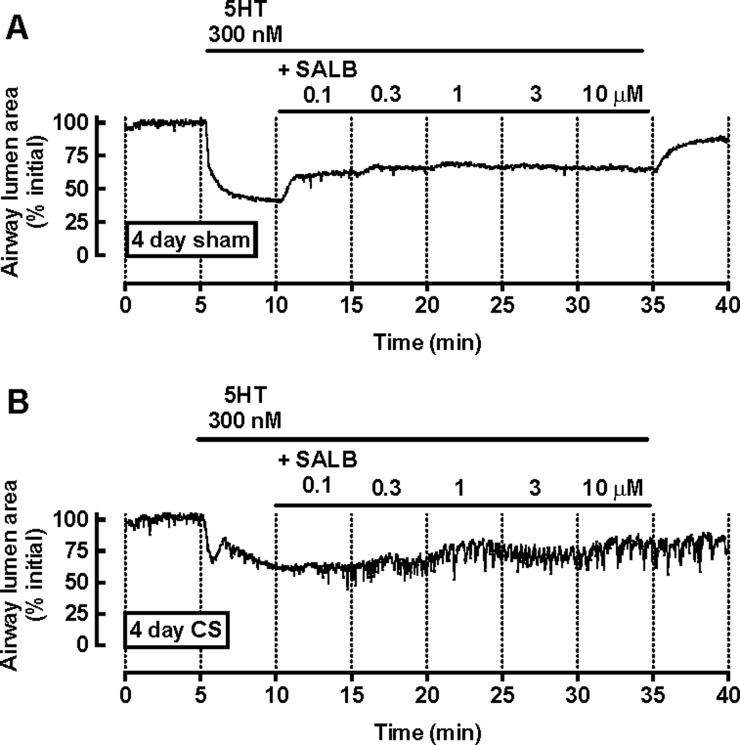
SALB-mediated relaxation is decreased following CS exposure Mice were exposed to room air (sham) or CS for 4 days before preparation of PCLS. Representative traces from (**A**) sham- and (**B**) CS-exposed mice show a frame-by-frame analysis (0.5 Hz) of changes in airway lumen area with time in the presence of 300 nM 5HT and increasing concentrations of SALB.

**Figure 2:**
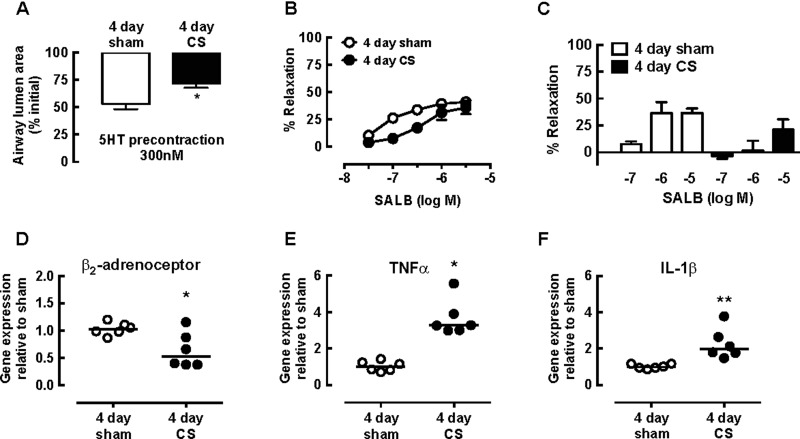
Decreased SALB-mediated relaxation following CS exposure is associated with decreased β-adrenoceptor expression and increased cytokine expression (**A** and **B**) Airways in PCLS from sham- and CS-exposed mice were exposed to 300 nM 5HT prior to cumulative additions of SALB. (**A**) Submaximal pre-contraction to 5HT, expressed as % initial airway lumen area and (**B**) % relaxation to SALB. Data are expressed as mean ± S.E.M. (*n*=4 per group, *n* represents one slice per mouse, * *P*<0.05 unpaired *t* test). (**C**) Airways in separate PCLS from sham- and CS-exposed mice were exposed to 300 nM 5HT prior to addition of single concentrations of SALB. Data are expressed as mean ± S.E.M. (*n*=3–7 per group). (**D**–**F**) Lungs from sham and CS-exposed mice were harvested on day 5. All lungs were homogenized and RNA was extracted using a Qiagen kit. Following conversion of RNA to cDNA, quantitative real-time PCR was performed using Taqman primers for (**D**) β_2_-adrenoceptor (**E**) TNFα and (**F**) IL-1β. Data were analysed by the ΔΔ*C*_T_ method, using *18S* as the control gene and shown as median and individual values (*n*=6 per group) * *P*<0.05 Mann–Whitney non-parametric test compared with PBS.

The potential influence of CS on dilator responsiveness in PCLS was assessed. We have recently identified altered contractile responses to 5HT after 4 days CS exposure [18]. As reported above, 4 days CS exposure led to a decrease in body weight (final weight–day 5: sham 24.9±0.7; CS 22.6±0.2, *P*<0.05, unpaired *t* test). To extend these findings, airways were pre-contracted with 300 nM 5HT, prior to addition of SALB (0.1–10 μM) (Figures 1 and 2).

Representative traces in PCLS from sham- and CS-exposed mice show that SALB induced concentration-dependent partial relaxation (Figure 1). Pre-contraction with 300 nM 5HT was lower in the CS group, with 5HT eliciting a biphasic contraction (% initial airway lumen area: sham 53.1±4.8; CS 71.8±3.9, *P*<0.05, unpaired *t* test) (Figures 1 and 2A). Relaxation to SALB was maximal at 3 μM in both groups (% relaxation: sham 40.9±7.9; CS 35.9±6.2) with ∼ 5-fold lower potency following CS (Figures 1 and 2B). In PCLS from CS-exposed mice, relaxation responses were transient and not well maintained over the concentration range tested (Figure 1B).

Relaxation responses to individual concentrations of SALB (0.1 μM, 1 μM and 10 μM) were tested in separate PCLS from both groups, to determine whether cumulative addition had contributed to the loss of β_2_-adrenoceptor-mediated relaxation following CS exposure. The level of submaximal pre-contraction to 5HT was consistent across all slices (results not shown). Relaxation responses to SALB in individual slices were similar to those seen at the same concentration in cumulative concentration–response curves (% relaxation to 10^−6^ M SALB: sham in individual PCLS 36.7±10.0; sham from cumulative curves 39.2±8.3, *P* > 0.05, unpaired *t* test). Relaxation responses in individual PCLS from CS-exposed mice were even lower than when added cumulatively (% relaxation to 10^−6^ M SALB: CS individual 1.8±9.0; CS cumulative 31.2±9.0) (Figure 2C).

This loss of dilator responsiveness was associated with ∼40% reduction in β_2_-adrenoceptor expression in the lungs of mice following 4 days CS exposure (Figure 2D). Both TNFα and IL-1β expression were increased ∼4-fold and ∼2-fold respectively (Figures 2E and 2F).

### CS exposure and Mem71 infection *in vivo* alters body weight

To assess the effect of this prior CS exposure on responses to influenza A virus infection, mice were treated with CS (or air for sham) for 4 days followed by 10^4.5^ PFU of Mem71 (H3N1) (or PBS) on day 5, then culled 7 days post-infection (i.e. on day 12 of the experimental protocol). In a similar model, mice treated with CS followed by Mem71 had sustained cellular inflammation in the lung over this period despite resolution of viral infection by day 10 [21,24]. In the present study, body weights were recorded daily, and PCLS were prepared on day 12 to assess constrictor responses to MCh and 5HT, and dilator responses to SALB and ISO.

**Figure 3:**
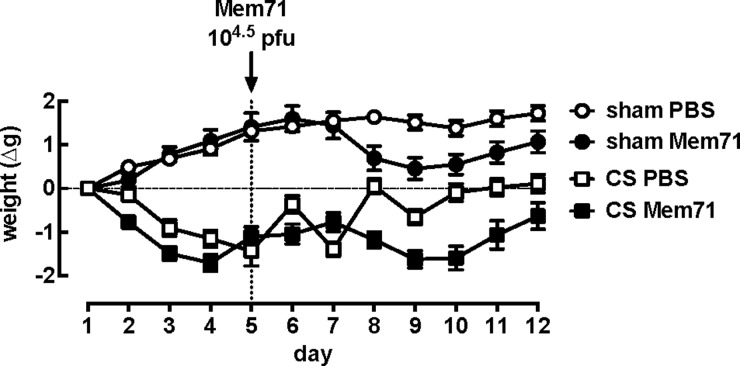
Mouse weights from sham or CS mice treated with PBS or Mem71 over the 12 day protocol Mice were exposed to air (sham) or CS for 4 days. On day 5, mice were administered 30 μl of PBS or 10^4.5^ PFU of Mem71 intranasally. All mice (sham PBS, CS PBS, sham Mem71 and CS Mem71) were weighed daily at 8:45 hours until day 12 (7 days post-infection). Data are expressed as mean ± S.E.M. as a change in weight from day 1 (*n*=10 per group).

Sham mice gained weight over the first 4 days period, whereas CS mice lost ∼1.5 g (weight: day 1–CS PBS 22.0±0.32 g; day 4–CS PBS 20.8±0.4 g, *P*=0.0001) (Figure 3). Following intranasal administration of PBS on day 5, sham PBS mice maintained their weight over the subsequent 7 days period. After cessation of CS exposure on day 5, CS PBS mice initially gained weight, but subsequently lost weight on day 7 prior to regaining their initial weight by day 12. Following intranasal administration of Mem71 on day 5, both sham Mem71 and CS Mem71 groups lost weight, with a peak weight loss occurring between days 3 and 5 post-infection. These mice then gained weight over days 6–7 post infection, but did not reach the same weight as the sham PBS controls (day 12 weights (g): sham PBS 25.6±0.6; CS PBS 22.1±0.4; sham Mem71 22.5±0.4; CS Mem71 20.5±0.4; *P*<0.0001 one-way ANOVA).

### CS exposure and Mem71 infection *in vivo* impairs dilator responses in mouse small airways *ex vivo*

Prior to assessment of bronchodilator responsiveness, constrictor responses to MCh and 5HT were compared in PCLS from sham PBS, CS PBS, sham Mem71 and CS Mem71 treated mice. The potency of both agonists was similar in all groups (Supplementary Figure S1). The maximum responses to both constrictor agents tested were similar between sham PBS and CS PBS groups. Increased maximal contraction to MCh was seen in sham Mem71 compared with sham PBS (*P*<0.05) and 5HT contraction tended to increase in CS Mem71 groups respectively (Supplementary Figure S1). These changes were not associated with differences in mRNA expression of M_2_, M_3_ or 5HT2A (results not shown).

**Figure 4:**
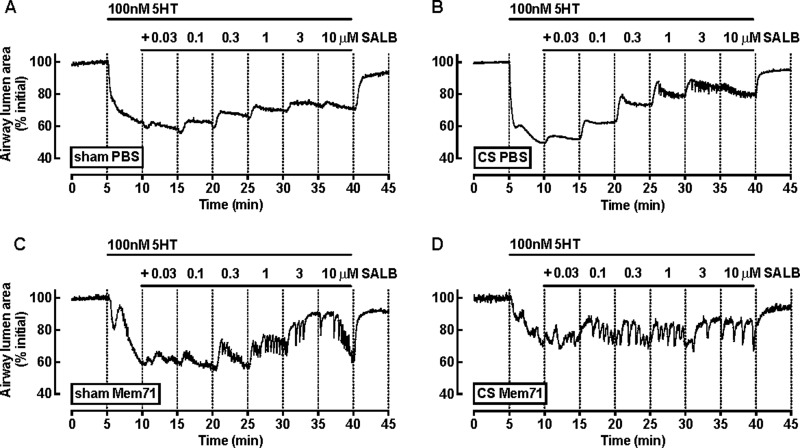
SALB-mediated relaxation is decreased following CS Mem71 exposure PCLS from (**A**) sham PBS, (**B**) CS PBS, (**C**) sham Mem71 and (**D**) CS Mem71 were prepared as described above. Representative traces show a frame-by-frame analysis (0.5 Hz) of changes in airway lumen area with time in the presence of 100 nM 5HT and increasing concentrations of SALB.

**Figure 5:**
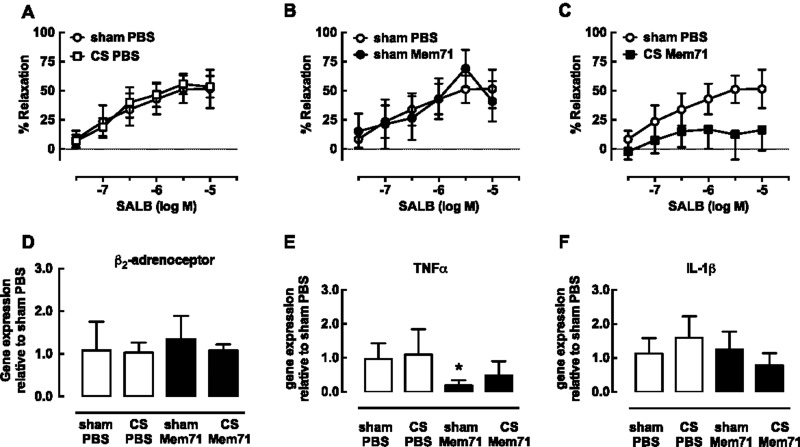
Decreased SALB-mediated relaxation following CS Mem71 exposure is not associated with altered β-adrenoceptor or cytokine expression (**A**–**C**) Airways in PCLS were exposed to 100 nM 5HT prior to cumulative additions of SALB. % Relaxation to SALB in sham PBS (*n*=3) was compared with (**A**) CS PBS (*n*=7), (**B**) sham Mem71 (*n*=4) and (**C**) CS Mem71 (*n*=9), where *n* represents one slice per mouse. Data are expressed as mean ± S.E.M. (**D**–**F**) Lungs were harvested and homogenized on day 12 and RNA was extracted using a Qiagen kit. Following conversion of RNA to cDNA, quantitative real-time PCR was performed using Taqman primers for (**D**) β_2_-adrenoceptor (**E**) TNFα and (**F**) IL-1β. Data were analysed by the ΔΔC_T_ method, using *18S* as the control gene and shown as median and interquartile range (*n*=6 per group) * *P*<0.05 Kruskall–Wallis one-way ANOVA with Dunn's *post-hoc* test compared with sham PBS.

Bronchodilator responses to SALB and ISO were then tested following submaximal pre-contraction with 5HT (Figures 4 and 5). In PCLS from sham PBS mice, 5HT induced a monophasic response (∼40% reduction in airway lumen area) (Figure 4A). The pattern of the pre-contraction to 5HT was altered in all other treatment groups. Small airways in PCLS from CS PBS (Figure 4B) and sham Mem71 (Figure 4C) mice had biphasic 5HT contractions, whereas the contraction to 5HT in the CS Mem71 group was rapid and not sustained, followed by a more slowly developing contraction (Figure 4D). However, similar reductions in airway area were achieved prior to exposure to SALB (not significant, *P* > 0.05, one-way ANOVA).

Although SALB induced concentration-dependent relaxation in all groups, the pattern of relaxation differed (Figure 4). Relaxation to SALB in PCLS from CS PBS mice was greatest within the first minute of exposure (Figure 4B), however this waned over time to reach a maximum of ∼50% at 10 μM levels of relaxation, similar to sham PBS mice (Figures 4A and 5A). In PCLS from sham Mem71 mice, relaxation was very transient and not well maintained, with a maximum relaxation response to 3 μM SALB and re-contraction evident at 10 μM SALB (Figures 4C and 5B). The transient and not well maintained relaxation responses to SALB in PCLS from CS Mem71 mice were more pronounced than in sham Mem71 or CS PBS mice. Relaxation was not sustained at any of the concentrations tested (Figures 4D and 5C) and was the lowest in response to 10 μM SALB of all groups (sham PBS 51.6±16.5; sham Mem71 41.1±17.2; CS PBS 53.5±9.4; CS Mem71 15.3±20.2) (Figures 5A–5C).

There were no differences in β_2_-adrenoceptor measured in whole lung lysates from all four groups (Figure 5D). However, TNFα mRNA expression was decreased by 80% in lungs from sham Mem71 mice, with CS PBS and CS Mem71 groups similar to sham PBS (Figure 5E). IL-1β mRNA expression was unchanged (Figure 5F).

In separate PCLS prepared from all groups, airways were pre-contracted with 5HT prior to ISO (Supplementary Figure S2). Relaxation was also the lowest in the CS Mem71 group with the maximum relaxation at 0.3 μM ISO (% relaxation: sham PBS: 74.3±19.8, CS Mem71 56.9±14.8).

## DISCUSSION

The present study has examined airway reactivity in PCLS prepared from mice exposed to CS (4 days) alone or mice exposed to CS and then infected with influenza A virus (Mem71). The major findings were that dilator responses to SALB were impaired by CS exposure alone, and almost completely abolished when CS exposure was followed by Mem71 infection. The reduction in relaxation in CS-exposed mice was associated with reduced β_2_-adrenoceptor expression and increased TNFα and IL-1β expression after acute CS exposure. However, the impaired bronchodilator responses in CS and influenza-infected mice were not associated with changes in β_2_-adrenoceptor, TNFα and IL-1β expression, suggesting additional mechanisms at play following viral infection. This reduction in β_2_-adrenoceptor-mediated relaxation is consistent with the loss of clinical responsiveness to dilator therapy in cigarette smokers and in AECOPD.

Respiratory viruses represent a large burden on our healthcare system, particular in patients with underlying lung diseases such as asthma and COPD, when their symptoms are exacerbated. Although research in the field has been focused around the immunological changes caused by viruses, the functional effects of viral infection on the airways, particularly in the distal lung, are not well characterized. However, clinical data have shown the viral infection induces airway hyperresponsiveness (AHR) to MCh in both healthy subjects and people with asthma and COPD [25,26] and AHR to histamine in asthmatic subjects following influenza A virus infection [27]. It has been established that viruses can alter airway reactivity through the production of immunoglobulin E (IgE), damage to the epithelium, increased inflammatory cytokine release and enhanced mediator release [28].

Bronchodilator therapy may be ineffective in patients with lung diseases who are exposed to viral infections. The most common reliever drug, SALB, a partial agonist at β_2_-adrenoceptors, dilates airways through increasing levels of cAMP. Diminished responsiveness to the β-adrenoceptor agonist ISO has been demonstrated following a viral upper respiratory tract infection, with the cAMP response greatest in healthy controls, slightly decreased in asthmatics and dramatically reduced in asthmatics with viral infection [29,30].

The aim of the present study was to establish whether a similar loss of β-adrenoceptor responsiveness seen clinically occurs in mouse models of CS exposure and combined CS and influenza A virus exposure. More importantly, we were able to evaluate the contribution of the small airways to this process by using PCLS. There is evidence in the literature that the magnitude and type of inflammatory response to CS and influenza A virus infection in mice is dependent on the strain of mouse and influenza A virus used. For the purpose of our study, we used our well characterized models in BALB/C mice which have a robust inflammatory response to CS compared with C57BL/6 mice [22]. Although C57BL/6 mice are more susceptible to virus infection due to a Th-1 driven immune response [31–33] and the production of type-1 interferons [34], inflammation following CS in this strain is limited [22]. In addition, an exacerbated viral response following virus and CS-exposure is seen in BALB/C but not in C57BL/6 mice [21,34,35].

The impact of CS and CS+ influenza A virus infection on body weight was assessed prior to airway reactivity experiments. We confirmed the pattern recently observed in CS- and influenza A virus-treated mice over the 12 days treatment period [21]. However, by measuring weight daily, the present study has also identified that in addition to CS alone causing weight loss, there was a biphasic weight gain when CS exposure was followed by a single PBS administration (CS PBS group). One explanation for the transient weight gain on day 6 is that smoking cessation and PBS treatment reduces CS-associated loss of appetite and/or dehydration. The subsequent loss of weight on day 7 may reflect a response to CS withdrawal, whereas the increased weight on day 8 may be due to appetite recovery and fluid intake as the effects of the CS are wearing off by this time, resulting in restored body weights by days 9–12 [36].

To assess CS- and influenza A virus-induced changes on airway reactivity, we have utilized the PCLS technique. One of the major advantages of this approach is that interactions between the airways and surrounding parenchyma remain intact when measuring contraction and relaxation [37,38]. Previous studies using mice treated with allergen to induce AHR *in vivo* have shown variable effects on *in vitro* responses to MCh in PCLS, which may reflect differential effects of these models on airway wall remodelling [39–41]. We do not anticipate gross structural changes following *in vivo* exposure to CS and/or influenza A virus in the present study, as similar models have shown no structural changes to CS and/or influenza A virus model culled at peak viral infection [21,22], and the acute exposure and resolution of inflammation by day 7 post-infection [24]. However, CS or its components have been shown to induce cellular toxicity to a range of cell types including bronchial epithelial cells and airway smooth muscle cells [42–44]. Assessment of reactivity day 7 post-infection (during resolution of inflammation) was chosen to assess whether CS-induced changes following 4 days of exposure [18] would be maintained, and whether changes in reactivity are evident even when viral load has been reduced.

Both 5HT and MCh increase both calcium oscillations and calcium sensitivity pathways to cause airway smooth muscle contraction. However, we have recently shown that 4 days CS exposure selectively altered airway contraction in PCLS to 5HT but not MCh, associated with down-regulation of ryanodine receptor expression [18]. In the present study, it was not surprising that contraction to MCh in CS PBS mice was unchanged when measured after 7 days in the absence of CS exposure. However, the normalized response to 5HT suggests restoration of normal contraction within 8 days. Interestingly, 7 days after sham Mem71 treatment, *in vitro* contraction to MCh was increased despite the absence of significant viral load [24]. *In vivo* AHR to MCh was not observed following respiratory syncytial virus (RSV) infection in mice [45], but the present finding is consistent with AHR following infection in the clinical setting [46] and also following parainfluenza type-3 virus infection in guinea pigs, associated with decreased production of nitric oxide post-infection [47]. This mechanism is unlikely to be underlying the increased response to MCh following Mem71 infection, as the *in vitro* perfusion of drugs across the PCLS would limit any influence of decreased production of epithelial-derived relaxing factors. However, although the mRNA expression of M_2_ and M_3_ receptors was unchanged, protein levels or receptor signalling may be altered, as shown in guinea pigs treated with parainfluenza where M_2_ dysfunction was seen [48]. In addition, the increased contraction to MCh in sham Mem71 mice, but not CS Mem71 mice, remains to be explained.

Following 4 days CS exposure, SALB-mediated relaxation was impaired both in potency and the ability to maintain a sustained relaxation. This was associated with β_2_-adrenoceptor down-regulation, and increased inflammatory cytokines, TNFα and IL-1β, shown to decrease β-adrenoceptor responsiveness in airway smooth muscle *in vitro* [49,50]. It is possible that the decreased potency of SALB in cumulative concentration–response curves in PCLS from CS-treated mice underestimated the effects of CS exposure. These airways were pre-contracted to a lesser extent than sham controls and it is well established that this would increase SALB potency [23]. Consistent with this, greater inhibition of SALB-mediated relaxation was seen in individual PCLS with matched pre-contraction after CS exposure. Under these conditions, the influence of previous exposure to lower concentrations of SALB within the same slice was removed, suggesting that there is not greater desensitization of the β-adrenoceptor following CS exposure. Of note, relaxation to SALB measured in PCLS prepared 7 days after cessation of CS exposure was restored, and was associated with normalized expression of the β_2_-adrenoceptor, TNFα and IL-1β.

One of the crucial findings of the present study was the loss of β-adrenoceptor responsiveness measured at day 7 post-infection. At this time point, these mice have almost no virus in the lung and the inflammatory response to the virus has subsided [36]. SALB-mediated relaxation in PCLS from both sham Mem71 and CS Mem71 mice was not well sustained across the full concentration range tested, and markedly impaired in the combined model where transient and not well maintained responses to SALB were evident, even at low concentrations. These combined findings indicate that impaired airway smooth muscle relaxation following CS exposure alone can be restored; viral infection alone can also reduce responsiveness to SALB, and that viral infection after CS exposure can exacerbate the loss of dilator sensitivity even when exposure to CS is ceased.

In both sham Mem71 and CS Mem71 groups, β_2_-adrenoceptor expression was unchanged, and TNFα and IL-1β were not increased, so the reduced responsiveness to SALB may be due to alternative viral-driven mechanisms that could include decreased production or loss of sensitivity to cAMP, increases in phosphodiesterase activity to reduce availability of cAMP, or increased activation of mechanisms driving contraction to oppose SALB-mediated relaxation. Since relaxation to the β_1_/β_2_-adrenoceptor agonist ISO was only slightly decreased in CS Mem71 treated mice relative to sham PBS, changes in the β_2_-adrenoceptor itself are also possible, with CS and Mem71-induced changes in the conformational state of the receptor to cause decreased signalling downstream.

In summary, this is the first study to demonstrate impaired β-adrenoceptor sensitivity of small airways in PCLS from mouse models of CS and/or influenza A virus infection. Although reduced SALB-mediated relaxation could be restored following cessation of CS exposure, subsequent viral infection abolished any sustained relaxation to the β_2_-adrenoceptor agonist. Future studies at day 3 post-infection, when inflammation and viral load are at their peak, are warranted to define the onset of mechanisms contributing to this loss of dilator sensitivity. The present study provides a validated model to assess novel bronchodilator agonists, which in combination with new antiviral medication may provide relief for lung disease patients with viral infections where their current therapy is ineffective.

## Abbreviations

AECOPD: acute exacerbations of COPD
AHR: airway hyperresponsiveness
COPD: chronic obstructive pulmonary disease
CS: cigarette smoke
HBSS: hanks balanced salt solution
5HT: serotonin (5-hydroxytryptamine)
IL-1β: interleukin-1β
ISO: isoprenaline
MCh: methacholine
PCLS: precision cut lung slices
PFU: plaque forming units
SALB: salbutamol
TNFα: tumour necrosis factor α
